# Paternity tests support a diallelic self‐incompatibility system in a wild olive (*Olea europaea* subsp. *laperrinei*, Oleaceae)

**DOI:** 10.1002/ece3.5993

**Published:** 2020-02-05

**Authors:** Guillaume Besnard, Pierre‐Olivier Cheptou, Malik Debbaoui, Pierre Lafont, Bernard Hugueny, Julia Dupin, Djamel Baali‐Cherif

**Affiliations:** ^1^ EDB UMR 5174 CNRS‐IRD‐UPS Université Paul Sabatier Toulouse cedex France; ^2^ CEFE, Univ Montpellier, CNRS, EPHE, IRD, Univ Paul Valery Montpellier 3 Montpellier France; ^3^ Laboratoire de Recherche sur les Zones Arides USTHB/ENSA Alger Algeria

**Keywords:** diallelic self‐incompatibility system, microsatellites, *Olea europaea* L., Oleaceae, paternity

## Abstract

Self‐incompatibility (SI) is the main mechanism that favors outcrossing in plants. By limiting compatible matings, SI interferes in fruit production and breeding of new cultivars. In the Oleeae tribe (Oleaceae), an unusual diallelic SI system (DSI) has been proposed for three distantly related species including the olive (*Olea europaea*), but empirical evidence has remained controversial for this latter. The olive domestication is a complex process with multiple origins. As a consequence, the mixing of *S*‐alleles from two distinct taxa, the possible artificial selection of self‐compatible mutants and the large phenological variation of blooming may constitute obstacles for deciphering SI in olive. Here, we investigate cross‐genotype compatibilities in the Saharan wild olive (*O. e.* subsp. *laperrinei*). As this taxon was geographically isolated for thousands of years, SI should not be affected by human selection. A population of 37 mature individuals maintained in a collection was investigated. Several embryos per mother were genotyped with microsatellites in order to identify compatible fathers that contributed to fertilization. While the pollination was limited by distance inside the collection, our results strongly support the DSI hypothesis, and all individuals were assigned to two incompatibility groups (G1 and G2). No self‐fertilization was observed in our conditions. In contrast, crosses between full or half siblings were frequent (ca. 45%), which is likely due to a nonrandom assortment of related trees in the collection. Finally, implications of our results for orchard management and the conservation of olive genetic resources are discussed.

## Graphical abstract

An unusual diallelic self‐incompatibility (SI) system has been proposed for distantly related Oleaceae lineages, but empirical evidence has remained controversial for the olive tree. We thus analyzed the SI system of the Laperrine's olive based on realized matings through parentage analysis. All individuals were assigned to two incompatibility groups (G1 and G2) supporting the diallelic self‐incompatibility system in this taxon.

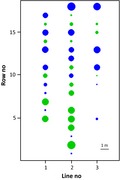

## 1 INTRODUCTION

Self‐incompatibility (SI) is the main mechanism that prevents self‐fertilization and promotes outcrossing in flowering plants (Taiz, Zeiger, Moller, & Murphy, 2015). A few, independent evolution events of SI have been documented, and the self‐incompatible lineages concerned (ca. 40% of angiosperms) belong to distantly related families, such as Brassicaceae, Rosaceae, or Solanaceae (Igic, Lande, & Kohn, 2008; de Nettancourt, 1977; Taiz et al., 2015). In addition to preventing self‐fertilization, SI can also act as a barrier between individuals sharing the same SI phenotype and, thus, influences pollen‐mediated gene flow by limiting compatible matings (Bateman, 1952). Moreover, by interfering with plant production and breeding, SI is a major obstacle for constant fruit production in crop species (Sassa, 2016), as well as for the breeding of new cultivars and the development of inbred lines (Matsumoto, 2014). Deciphering the SI system of plants is therefore of great interest in agronomy, horticulture, and forestry.

In the Olive tribe (Oleeae), the SI system has been recently subject to investigations in the genera *Phillyrea*, *Fraxinus*, and *Olea* (Saumitou‐Laprade et al., 2010; Saumitou‐Laprade, Vernet, Vekemans, Billiard, et al., 2017; Saumitou‐Laprade et al., 2018; Breton et al., 2014; Vernet et al., 2016). In the regular SI system, negative frequency‐dependent advantage promotes the emergence of new *S*‐alleles and their maintenance across speciation (Igic et al., 2008; Wright, 1939). In Oleeae on the other hand, an unusual, diallelic SI system (DSI) has been proposed for those three distantly related lineages (Saumitou‐Laprade et al., 2010) that have diverged from a common polyploid ancestor during the Eocene (Olofsson et al., 2019; Taylor, 1945; Wallander & Albert, 2000). This homomorphic DSI system is composed of an *S*‐locus with two alleles, *S2* and *S1* (with *S2* dominant over *S1*), that produces two incompatibility groups, G1 (*S2S1*) and G2 (*S1S1*; Billiard et al., 2015). Further, in Oleeae, the reciprocity of compatibilities between *Phillyrea angustifolia* L., *Fraxinus ornus* L., and *Olea europaea* L. suggests identical recognition specificities between these three taxa (Saumitou‐Laprade, Vernet, Vekemans, Billiard, et al., 2017; Vernet et al., 2016). The DSI system could, thus, be conserved among Oleeae species, and it was also suggested to be linked to the evolution and maintenance of androdioecy in *Phillyrea* and *Fraxinus* section* Ornus* (Billiard et al., 2015; Husse, Billiard, Lepart, Vernet, & Saumitou‐Laprade, 2013; Van de Paer, Saumitou‐Laprade, Vernet, & Billiard, 2015). While the presence of a DSI system in *Phillyrea* and *Fraxinus* is recognized by the scientific community (Pannell & Voillemot, 2015), the doubt remains in the cultivated olive tree (*O. e.* subsp. *europaea*) because the few studies that investigated the mating system in the species present conflicting results (e.g., Farinelli et al., 2018; Saumitou‐Laprade, Vernet, Vekemans, Castric, et al., 2017).

Currently, there are two main methods that are used to determine compatibility between olive varieties: (a) the comparison of fruit sets from crosses under bags to fruits sets from self‐ or free‐pollination (e.g., Farinelli, Breton, Famiani, & Bervillé, 2015) and (b) the observation of the presence or absence of pollen‐tubes converging toward the style after pollination (so‐called stigma tests; Saumitou‐Laprade, Vernet, Vekemans, Billiard, et al., 2017). Based on fruit set observations, asymmetric cross‐compatibilities were observed among studied cultivars leading some authors to propose a sporophytic model involving six *S*‐alleles with different dominance relationships (Breton et al., 2014). However, contradictory results have been reported for cross‐compatibilities between varieties depending on their location or year of study (Bartolini & Guerriero, 1995), such discrepancies being sometimes explained by a partial self‐compatibility system influenced by environmental conditions (Ateyyeh, Stosser, & Qrunfleh, 2000; Bradley & Griggs, 1963; Moutier, 2000). In addition to those factors, methodological issues, such as uncertainty around identity of varieties (with the possible vegetative propagation of SI mutants), pollen contamination, and neglection of stigma receptivity (with large phenological variations among cultivars and years), could also all explain such inconsistencies (Díaz, Martín, Rallo, & de la Rosa, 2007; Mookerjee, Guerin, Collins, Ford, & Sedgley, 2005; Saumitou‐Laprade, Vernet, Vekemans, Castric, et al., 2017). In contrast, methods based on both intra‐ and interspecific stigma tests on a representative sample of olive cultivars indicated that *Olea*, *Phillyrea,* and *Fraxinus* share the same DSI system (Saumitou‐Laprade, Vernet, Vekemans, Billiard, et al., 2017). Some authors have, however, expressed doubts about these results due to strong divergences with their previous studies (i.e., asymmetry of incompatibilities in reciprocal crosses and pollen germination not sustaining DSI; Breton, Koubouris, Villemur, & Bervillé, 2017; Farinelli et al., 2018). Such disagreeing findings, thus, call for accurate SI tests on a homogeneous genetic pool, ideally on natural populations of *O. europaea*. Considering the two alternative hypotheses, distinct patterns of cross‐compatibility between individuals are expected within a population, with only two groups of reciprocal compatibility under the DSI hypothesis, while a more complex pattern should be observed for the alternative multigroups hypothesis implying some nonreciprocal compatibilities among individuals or groups of incompatibility. Under this latter hypothesis, incompatibility groups could thus be difficult to define, and the required number of observations will increase with the number of *S*‐alleles involved.

In this work, we aimed to phenotype the SI system of the wild Laperrine's olive [*O. europaea* subsp. *laperrinei* (Batt. & Trab.) Cif.] using paternity tests. The Laperrine's olive is endemic to the Saharan mountain ranges (above 1,200 m), and most of its populations have been isolated from the Mediterranean basin long before olive domestication (excepted in the Tassili'n Ajjer; Baali‐Cherif & Besnard, 2005; Besnard et al., 2013). Indeed, investigating the SI system in this wild subspecies is relevant because it should not be affected by human‐related selective effects or recent admixture (Besnard, Anthelme, & Baali‐Cherif, 2012). Moreover, because of the relative synchronization of flowering between trees, the study of SI in the Laperrine's olive is not affected by phenological variations like in the cultivated olive. To phenotype, then, the SI system in this subspecies of wild olive, we used paternity tests with microsatellites markers. A previous study demonstrated that the use of paternity tests is an efficient approach to identify cross‐compatibilities between individuals in this taxon (Besnard, Baali‐Cherif, Bettinelli‐Riccardi, Parietti, & Bouguedoura, 2009). It may even be more appropriate on an artificial, open‐pollinated tree population maintained in a collection. A localized pollen cloud associated to an isolation from external pollination should indeed greatly facilitate the identification of fathers. To avoid methodological problems (e.g., pollen contamination, identity of genotypes…), we thus analyzed the SI system based on realized matings through parentage analysis from fruits collected on the 37 mature trees of a Laperrine's olive collection. Several embryos per individual were genotyped with microsatellite markers in order to identify pollen donors. Our paternity analyses strongly support the existence of two reciprocally compatible groups and are fully consistent with the DSI system proposed by Saumitou‐Laprade, Vernet, Vekemans, Billiard, et al. (2017). In addition, the use of a few controlled crosses between Mediterranean and Laperrine's olives allows establishing correspondence between the two groups defined in the Laperrine's olive and the G1 and G2 groups observed in cultivated olives and *P. angustifolia*. On open‐pollinated trees, other features of the mating system of the Laperrine's olive, as distance of pollination and variable paternal contribution, were also investigated and compared to previous studies conducted in natural populations. Implications of these results for orchard management, genetic improvement of the domesticated olive, and conservation of wild olive genetic resources are finally discussed.

## 2 MATERIALS AND METHODS

### 2.1 Plant material

The trees used in this study are maintained in a collection at the common garden of the “Plateforme des Terrains d'Experience du LabEx CeMEB,” (CEFE, CNRS) in Montpellier, France (Table S1). This collection has Laperrine's olives (51 individuals) and a few Mediterranean olives (16 individuals). It also includes trees of subspecies *maroccana* (three trees), *cerasiformis* (1), *cuspidata* (7), and hybrids (2), but all flowers of these trees were manually removed before blooming (in June 2018), to avoid crosses with studied trees of the collection.

Among the 51 Laperrine's olive, 40 come from the study of Besnard et al. (2009) and correspond to seedlings from eight mothers at four different localities in the Hoggar, Algeria (Adjellela, Akerakar, Tonget and Tin‐Hamor; Figure S1). The nomenclature used for these trees is the following: first, the name of the locality, followed by a first number giving the identity of the mother and a second number to distinguish its seedlings. For example, the individual named “Adjellela_10_S1” is the seedling number 1 from the tree numbered 10 located in the Adjellela population. Several trees of the collection have the same mother tree and correspond to full siblings or half siblings (Table S2). The presence of closely related trees may potentially reduce the father assignation power in paternity analyses because of a reduced genetic diversity among those genotypes. However, phenotyping SI in related individuals may give insights into the genetic inheritance of incompatibility and into the actual efficiency of SI in avoiding matings between closely related individuals. In addition, the collection was complemented with eight trees from the Tonget area (named “Tonget_A” to “Tonget_H”), one individual from Tin‐Hamor (“12_S1”), one individual from Tizouadj (“2_S1”), and one triploid tree from Hadriane propagated by cutting (Besnard & Baali‐Cherif, 2009).

All available knowledge on the parent identity of each Laperrine's olive tree of the collection is reported in Table S2. Thirty‐seven mature Laperrine's olive individuals flowered in 2018, representing the highest proportion of blooming trees since the establishment of the collection in 2011 (see Table S2). The Laperrine's olive blooms approximately 1 month later than cultivated olives (usually from the end of June to mid‐July); therefore, contribution of other pollen donors outside the collection is very unlikely. In 2018, even if mature individuals of Laperrine's olive did not start flowering the same day, they were synchronously blooming at the tenth of July. The collection could, thus, be seen as an isolated system, and the father of any seed was expected among the 37 flowering individuals.

The Mediterranean olives of the collection were also investigated to be compared to the Laperrine's olives. They represent both cultivated varieties (11 trees) and oleasters (5). Fifteen of these trees flowered in 2018 from the end of May to the beginning of June. Two varieties of this collection, “L4‐R15” and “Sabina [L4‐R12],” were previously phenotyped for their incompatibility group in 2014 with stigma tests (Saumitou‐Laprade, Vernet, Vekemans, Billiard, et al., 2017). These two trees were cross‐compatible and attributed respectively to groups named G1 and G2 (P. Saumitou‐Laprade and Ph. Vernet, personal communication). In addition, by comparing genetic profiles at nine loci (DCA01, DCA03, DC04, DCA05, DCA08, DCA09, DCA15, DCA18, and EMO03; see Data S1A), we identified three cultivated varieties in common with the study of Saumitou‐Laprade, Vernet, Vekemans, Billiard, et al. (2017): “Manzanilla de Sevilla [L4‐R11]” = “Oit1” (assigned to group G2), “Arbequina [L4‐R13]” = “Oit26” (group G1), and “Koroneiki [L4‐R14]” = “Oit52 (group G1).

While most inflorescences of Mediterranean and Laperrine's olives were open‐pollinated in 2018, a few controlled crosses between these two taxa were performed. Bags were placed before blooming (in mid‐May 2018) on one branch with at least ten inflorescences on ten Laperrine's olives and ten Mediterranean olives (Table S1). Pollen of the Laperrine's olive was collected in July 2015 on “Adjellela_10_S9.” It was conserved at −80°C in aluminum foil. This pollen was used to pollinate the Mediterranean olives at the end of May 2018. Similarly, pollen of cultivated olive varieties was collected in May 2018 and conserved at −80°C before to be used for pollination of the Laperrine's olive. At this stage of the study, we have almost no knowledge about the compatibility between trees of the collection. We thus mixed pollen of five varieties (“Manzanilla de Sevilla [L4‐R11],” “Koroneiki [L4‐R14],” “L4‐R17,” “L4‐R19,” and “Amygdalolia [L4‐R20]”) to increase the probability of cross success with the Laperrine's olive.

Fruits were collected at the end of October 2018. First, we collected those resulting from open pollination. For trees with a large fruit set, we collected about 100 seeds (25 on each side of the tree), while all fruits were collected when the fruit set was limited (<100 fruits). Then, we collected fruits resulting from controlled crosses. A total of 29 putatively hybrid fruits were obtained: 13 for “Koroneiki [L4‐R14],” five for “L4‐R19,” one for “L4‐R17,” two for “Adjellela_10‐S7,” seven for “Tin‐Hamor_1_S14,” and one for “Tin‐Hamor_1‐S4.”

### 2.2 DNA extraction and genotyping with microsatellites markers

DNAs were extracted with the BS15 DNA Plant extraction kit (Qiagen Biosprint 15), either from a leaf fragment (for each diploid individual of the collection, excluding five juveniles; Table S1) or from embryos (for the offsprings). Embryos were isolated from each seed as follow: We first removed the endocarp, and seeds were then deposited on paper humidified with deionized water during 12 hr before separating the embryo from the albumen. For each mother tree of Laperrine's olive, several embryos were genotyped in order to identify some, compatible fathers. Less than five seeds were however available for trees with a very limited set of seeded fruits [i.e., “Hadriane_2.1,” “Akerakar_3_S1” and “Tin‐Hamor_1_S1”; note that most fruits (>99%) of the triploid “Hadriane_2.1” were empty indicating a high level of abortion]. We thus genotyped between two and 30 embryos per Laperrine's olive mother tree [for a total of 455 embryos from 36 mother trees (on average, 12.6 ± 5.5 embryos/mother tree); Table S2; note that one flowering individual (“Tin‐Hamor_1_S8”) did not produce any fruits]. In *O. europaea*, a fruit usually contains one seed, but we observed a relatively high frequency (ca. 10%) of multiseeded fruits in the Laperrine's olive. A specific nomenclature was, thus, used in order to identify seeds sampled from the same fruit. For example, “Adjellela_10_S1‐1A” and “Adjellela_10_S1‐1B” are embryos from two different seeds from the same fruit.

In addition, we also analyzed three to four embryos per Mediterranean olive tree (for a total of 46 embryos from 15 mother trees) to determine a few cross‐compatibilities within subspecies *europaea*. Lastly, embryos from the 29 seeds obtained from controlled crosses were also analyzed to determine cross‐compatibilities between individuals of subspecies *europaea* and *laperrinei*.

Sixteen microsatellite loci (Arbeiter, Hladnik, Jakše, & Bandelj, 2017; Carriero, Fontanazza, Cellini, & Giorio, 2002; Salmona et al., 2020; Sefc et al., 2000; Table 1; Data S1) were used to characterize 71 mature diploid/triploid individuals of the CEFE collection (subspp. *europaea*, *laperrinei*, *cuspidata,* and hybrids) plus 32 embryos of Laperrine's olive and the 46 embryos of Mediterranean olive. We used PCR conditions described by Salmona et al. (2020). PCR products were diluted and multiplexed together with GenScan‐600 Liz (Applied Biosystems) in formamide. After denaturation at 96°C, fragments were separated on an ABI Prism 3730 DNA Analyzer (Applied Biosystems) at the Genopole platform of Toulouse. Allele size was determined with Geneious v.9.0.5 (Kearse et al., 2012). To reduce genotyping error, microsatellite alleles were read independently twice by two different persons (MD and GB). The ability of the markers to assign a father was assessed independently on the Laperrine's olive collection (45 diploid trees, excluding juveniles and the triploid “Hadriane_2.1”), and on the Mediterranean olives (16 trees) by calculating the probability of exclusion of each marker and the combined probability of exclusion for all the markers using CERVUS v.3.0.7 (Kalinowski, Taper, & Marshall, 2007; Marshall, Slate, Kruuk, & Pemberton, 1998). We then selected nine loci to analyze the 423 remaining embryos of Laperrine's olive and the 29 putative hybrids. These loci were chosen based on three criteria: their relatively high heterozygosity leading to a high father‐discriminating power, their readability facilitating their scoring, and the allele size range that allowed multiplexing all loci in the same electrophoresis run. Embryos of Laperrine's olive that could not be assigned to a single father with these nine loci (see below for the paternity analyses) were finally analyzed with additional loci. Based on the genetic profile of putative fathers, we choose between one and five additional loci to identify, when possible, the true father.

**Table 1:** Characteristics of the 16 microsatellite loci used and summary of their genetic variability in the Laperrine's olive collection (for 45 diploid trees) and Mediterranean olives (16 trees): number of alleles (Na), expected heterozygosity (*H*_E_), observed heterozygosity (*H*_O_) and nonexclusion probability (NEP) in the paternity analysis

Locus^a^	Laperrine's olive (45)					Mediterranean olive (16)				
	Allele size range	Na	*H*_E_	*H*_O_	NEP	Allele size range	Na	*H*_E_	*H*_O_	NEP
**DCA01** *(6‐FAM)*	221–267	12	0.885	0.851	0.248	203–267	9	0.667	0.688	0.550
**DCA03** *(HEX)*	224–244	7	0.725	0.617	0.510	228–252	10	0.903	0.875	0.253
**DCA04** *(AT550)*	135–165	10	0.781	0.830	0.413	133−189^b^	11	0.861	0.750	0.317
**DCA05** *(HEX)*	194–240	11	0.854	0.809	0.306	190–210	9	0.700	0.625	0.515
**DCA08** *(HEX)*	116–144	8	0.786	0.894	0.416	124–158	9	0.754	0.688	0.458
DCA09 *(HEX)*	165–189	6	0.577	0.574	0.687	159–203	15	0.919	0.938	0.217
**Nor‐12** *(6‐FAM)*	175–193	7	0.764	0.717	0.436	177−241^b^	10	0.883	0.875	0.285
DCA15 *(HEX)*	245–252	2	0.225	0.170	0.901	241–262	5	0.679	0.688	0.602
DCA18 *(HEX)*	150–180	9	0.802	0.773	0.393	158–182	10	0.895	0.938	0.263
EMO03 *(HEX)*	194–217	10	0.782	0.745	0.429	211–218	7	0.819	0.875	0.406
GAPU71A *(6‐FAM)*	213–221	4	0.588	0.638	0.686	207–221	4	0.337	0.313	0.822
Nor‐10 *(AT565)*	216–240	7	0.810	0.745	0.383	213–234	7	0.837	0.938	0.373
Nor‐13 *(AT565)*	108–114	3	0.423	0.468	0.789	111–132	5	0.768	0.625	0.484
Nor‐15b *(6‐FAM)*	97–113	5	0.478	0.511	0.744	91–123	10	0.875	0.875	0.302
Nor‐17 *(AT550)*	158–197	7	0.714	0.622	0.530	168–203	7	0.825	0.750	0.396
Nor‐11 *(AT565)*	169–197	9	0.757	0.674	0.466	167–185	6	0.653	0.625	0.630

Note

Markers in bold correspond to the nine selected loci used to characterize all embryos of Laperrine's olive, while loci EMO03, Nor‐13, Nor‐17, DCA18, and GAPU71A were additionally used on a few embryos when necessary to discriminate some putative fathers that are closely related. The 16 loci were used to characterize all embryos of Mediterranean olives.

a The fluorochrome used for each locus is indicated in parenthesis.

b Evidence for null alleles in progenies; NEP = nonexclusion probability.

### 2.3 Paternity analyses and identification of compatible matings

Paternity analyses were performed using CERVUS (Marshall et al., 1998). This software uses a likelihood‐based method. The most likely father is determined from the log‐likelihood ratios (LOD score) based on the genotypes of the offspring, known mother, and each candidate sire (including the mother itself as a putative father). If the LOD score is equal to zero, the supposed father is as likely to be the real father as a male randomly selected. When the LOD score is positive, the alleged father is more likely to be the real father than a male randomly selected. All putative fathers with a positive LOD score were identified by the program. The simulation parameters were the following: 100,000 simulated offsprings, 37 candidate diploid parents, 1 as the proportion of candidate fathers sampled (we assumed that all potential fathers are in the collection), 0.94 as the proportion of loci typed (this value was calculated by an allele frequency analysis implemented in CERVUS), 0.0001 as the proportion of loci mistyped (we assumed a very low rate of mistyping because the data were checked independently twice; and redone when necessary), and 7 to 14 loci as the minimum number of typed loci (depending on the minimum number of successfully characterized loci available for an embryo). Putative self‐fertilizations among analyzed seeds were also carefully checked (considering the possibility of nonfertilization; i.e., haploid seed). Furthermore, when three alleles were observed at several loci, it was considered that the embryo was likely triploid or aneuploid (Besnard & Baali‐Cherif, 2009). As CERVUS is not implemented to analyze such data of variable ploidy, we compared genetic profiles of each triploid/aneuploid embryo with their mother in order to identify alleles inherited from the father. Then, paternal alleles were used to manually identify all putative father(s) in the collection.

The number of distinct fathers detected according to the number of embryos analyzed was estimated based on our observations. For each mother tree, the mean number of distinct fathers identified, *n*_f_, was calculated for a given number of embryos, *K* (*K* varying from 2 to a maximum of 18 embryos). Based on the list of fathers assigned to embryos analyzed, *n*_f_ was estimated for a given mother at each *K* value using a random sampling without replacement of *K* fathers with 10,000 independent iterations, using the function “rrarefy” implemented in the “vegan” package v.2.5‐6 (Oksanen et al., 2019) in R (R Core Team, 2019). At each *K* value, we only considered all mother trees with at least *K* embryos analyzed. The *n*_f_ matrix finally allowed us to estimate a global mean number of distinct fathers with a 0.95 confidence interval at each *K* value. These data were used to reconstruct an accumulation curve using the package “ggplot2” v.3.2.1 (Wickham, 2016) in R.

Groups of cross‐(in)compatibility were researched by analyzing mating patterns among Laperrine's olive individuals. The matrix of successful mating (see Section 3; Table 2) was first coded and simplified as a square and symmetric binary matrix with a 1 in a given cell *i*,*j* if individual *i* produced at least one seed with paternity attributed to *j*, or if individual *j* produced at least one seed with paternity attributed to *i*, and a 0 otherwise. We then performed a factorial correspondence analysis (FCA) on this matrix in order to identifying groups of individuals (if any) that preferentially mate among themselves or with members of another group. FCA was conducted using the R package “ade4” v.1.7.13 (Dray, Dufour, & Chessel, 2007).

**Table 2:**
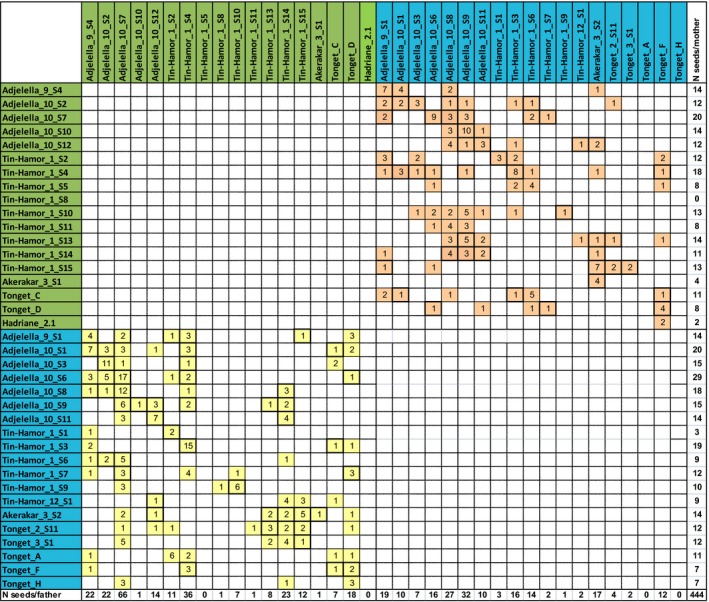
Representation of observed cross‐compatibilities among the Laperrine's olive collection after sorting individuals by cross‐incompatibility group: A (Blue; G2) and B (Green; G1), according to the FCA (Table S3)

Note

Numbers indicate the number of embryos for a given cross, an empty cell means no crosses happened. Mothers are in rows, fathers in columns. Pairs of reciprocal crosses are framed in bold line. A total of 444 embryos were assigned to a single father (see Table S4 for the unsorted table, also including unassigned embryos).

Lastly, paternity analyses were conducted on the few Mediterranean olive seeds and putatively hybrid seeds as described above. As cultivated varieties “L4‐R15” and “Sabina [L4‐R12]” were already assigned to the two incompatibility groups according to Saumitou‐Laprade, Vernet, Vekemans, Billiard, et al. (2017), these tests allowed us to link SI pattern observed in each subspecies and assign a putative incompatibility group to all studied trees.

### 2.4 Limitation of pollination efficiency by distance inside the Laperrine's olive collection

Finally, several features of the mating system, namely the mean distance of pollination, the relation between pollination and distance, and the differential distance of pollen dispersal between individuals, were described in the Laperrine's olive collection. An expected randomly distribution of distances was determined by sampling mother‐father pairs (with the function “sample” in R) regardless of distance but respecting cross‐compatibility and the number of observations, *n* (number of embryos assigned to a single father in our experiment). The mean of 1,000 random sampling of *n* pairs was then done to estimate an expected distribution without limitation by distance, which was compared with the observed distribution. For each mother, we compared the mean distance from the father between our observations and under a random process using a rank test (Mann–Whitney *U* test).

## 3 RESULTS

### 3.1 Microsatellite polymorphism, marker selection and father discrimination

The microsatellite dataset generated in this study is given in Data S1. All the 16 loci used to genotype the collection were polymorphic on both subspecies *laperrinei* and *europaea* but with variable level of diversity (Table 1). By investigating progenies, it was also possible to detect loci with null alleles (i.e., absence of a maternal allele on some embryos).

On the Laperrine's olive collection, no locus with null alleles was detected. The probability of nonexclusion given a known mother (i.e., the probability that an unrelated individual will not be excluded as a father) ranged from 0.248 (locus DCA01) to 0.901 (DCA15). The combined nonexclusion probability given a known mother (i.e., the average probability that the set of loci used will not exclude an unrelated individual as a father) reached 1.2e^−5^ for the 16 loci, and 3.8e^−4^ for the nine loci selected to characterize all embryos of Laperrine's olive.

On the Mediterranean olive collection, two loci [DCA4 and Nor‐12; this latter being homologous to DCA11 described in Sefc et al. (2000)] showed null alleles in the Mediterranean olive, each on two parents (Data S1). In addition, an excess of homozygosity is usually measured in the Mediterranean olive for these two loci (Sefc et al., 2000) as expected when null alleles are present. Paternity analyses were, thus, performed without DCA4 and Nor‐12 (that were further used to confirm father identification). For the 14 remaining loci, the probability of nonexclusion given a known mother ranged from 0.217 (DCA09) to 0.822 (GAPU71A). The combined nonexclusion probability given a known mother reached 5.1e^−6^ for these 14 loci.

### 3.2 Paternity analyses within the Laperrine's olive collection

#### 3.2.1 Father identification

The 32 embryos of Laperrine's olive first genotyped with the 16 markers (during the selection step of best loci) were all assigned with CERVUS to a single father of the collection. Then, the high cumulative exclusion probability of the nine selected loci allowed us to discriminate a single father for 365 of the 423 remaining embryos of Laperrine's olive. One additional embryo (“Adjelella_10_S6‐19”) corresponded to a hybrid between Laperrine's and a Mediterranean olive tree, but the pollen donor does not belong to the CEFE collection and so is unknown. For the 57 embryos assigned to at least two putative fathers of the collection with a high probability, the use of one to five additional loci made the identification of a single father possible for 48 of them [leading to single father assignation of 445 embryos on the 455 analyzed (97.8%), plus identification of one hybrid]. For the remaining nine embryos, two or four putative fathers were identified in our collection (respectively, for eight and one embryos; Data S1B), and the identification of the true progenitor was not possible even with all 16 markers. The inability to assign a single father for these embryos correspond, in all cases, to the nondistinction between full siblings (i.e., “Adjellela_10_S1, S2, S3, S6, S8, S9, and S12”; and “Tin‐Hamor_1_S7 and S15”). In addition, in eight cases, the mother was also a full sibling of the putative fathers.

#### 3.2.2 Paternal contributions

On average, we detected 4.83 ± 1.87 fathers/mother, from a minimum of one (in “Akerakar_3_S1” and “Hadriane_2.1” for which the number of embryos was limited to four and two, respectively) to a maximum of nine (for “Tin‐Hamor_1_S4”; Table 2). The mean number of distinct fathers identified in the Laperrine's olive collection depends on the number of analyzed embryos but does not follow a linear regression (Figure S2). On average, 5.2 distinct fathers are expected to be identified when genotyping 12 embryos. This value reaches six fathers for 18 embryos, meaning that increasing the embryo sampling by 50% is expected to increase by ca. 16% the number of observed compatible crosses.

On average, a tree pollinated 4.70 ± 3.65 mother trees, with a high heterogeneity in pollination contribution among the 37 mature individuals (Table 2). Four father trees (“TinHamor_1_S5,” “Tonget_A,” “Tonget_H,” and “Hadriane_2.1”) sired none of the embryos genotyped, while six sired only one embryo (in particular “Tin‐Hamor_1_S8” that did not produce any fruits). In contrast, “Adjellela_10_S7” is the father that sired the greatest number of embryos (66; 14.9% of assigned embryos). All trees (except the triploid “Hadriane_2.1”) that lowly or did not contribute as a father also produced a limited fruit set (<50 fruits; Table S2). It thus seems that their limited paternal contribution could be due to a reduced flowering. In contrast, the triploid status of “Hadriane_2.1” may reduce its reproductive success (e.g., due to abortion of pollen or sired embryos).

Multiseeded fruits represent 11.3% of the total number of fruits analyzed (46 of the 407 fruits, with 44 containing two seeds and two containing three seeds). Among the 46 fruits containing more than one seed, 29 were sired by the same father and 17 by different fathers. Among the two three‐seeded fruits, one contained three seeds sired by the same father, while the other contained three seeds sired by three different fathers.

#### 3.2.3 Relatedness between parents of seeds

**Figure 1:**
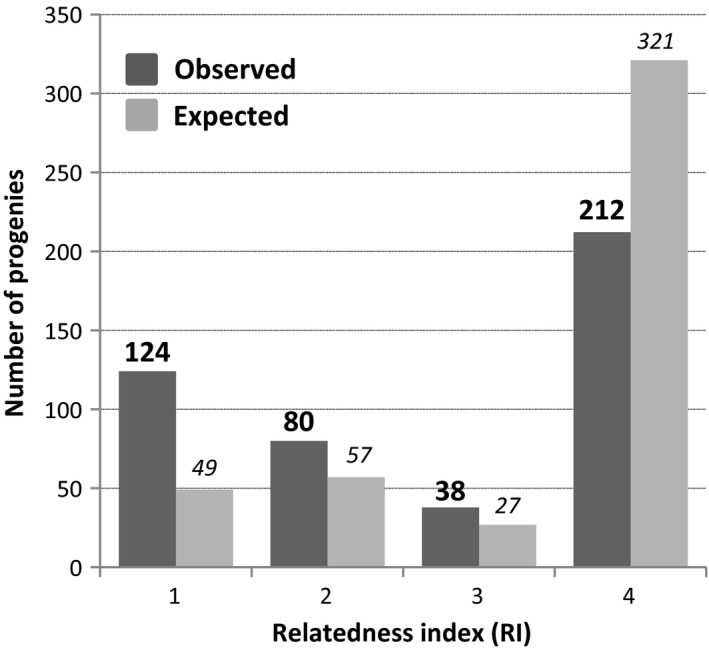
Level of relatedness between the mother and the father of the 454 analyzed progenies of Laperrine's olive (excluding the nonfertilized embryo). The relatedness was deduced from available knowledge on the parents of the Laperrine's olive trees of the CEFE collection (Table S1; Data S1B). 1 = crosses between full siblings; 2 = crosses between half siblings; 3 = crosses between individuals from the same population but not sharing a parent; 4 = crosses between individuals from distinct populations. Our observations were compared to expected levels of relatedness between compatible parents considering no limitation of pollination by distance and equal contribution of each parent. An excess of crosses between related individuals was observed, probably due to the nonrandom disposition of trees in the collection (i.e., individuals originating from the same population placed on the same lane; Table S1) and variable parental contribution of trees (Table S7)

As the parents of the Laperrine's olive trees in the CEFE collection are known for most trees, it was possible to assess the relatedness between parents of each seed (Figure 1). This analysis reveals that more than a quarter of analyzed embryos (27.3%) resulted from crossing between full siblings, while 17.6% were issued from crossing between half siblings. Less than half of the embryos (46.7%) came from crossing between parents originating from two different populations (Figure 1).

#### 3.2.4 Genotypic abnormalities of a few embryos

Four cases of triploid/aneuploid embryos were observed (“Adjelella_10_S12‐5,” “Akerakar_3_S2‐5,” and the two embryos obtained from the triploid mother “Hadriane_2.1”). Their genotypes are characterized by the presence of three alleles on one or two loci (Data S1A). In addition, the relative amplification of two alleles on other loci is also compatible with a triploid state (i.e., one allele twice more amplified than the other). The genetic characterization of these genotypes was repeated to insure the genotyping reliability. If we exclude the two “Hadriane_2.1” embryos, we thus observed two triploid/aneuploid embryos issued from crosses between diploid parents (among 453; ca. 0.4%).

Just one putative case of self‐fertilization (“Adjelella_9_S4‐6”) was identified by our paternity analysis, but the characterization of this embryo with the 11 heterozygous loci of the mother reveals only one allele at each locus. The probability to fix at random 11 loci by self‐fertilization is very low (0.5^11^ = 1/2048) indicating the embryo is very likely either haploid or di‐haploid, involving no fertilization event. So, based on these results of the year 2018, there was no evidence of self‐fertilization in seeds issued from open pollination in the Laperrine's olive.

### 3.3 Identification of groups of cross‐incompatibility

#### 3.3.1 Distinction of cross‐incompatibility groups in the Laperrine's olive collection

**Figure 2:**
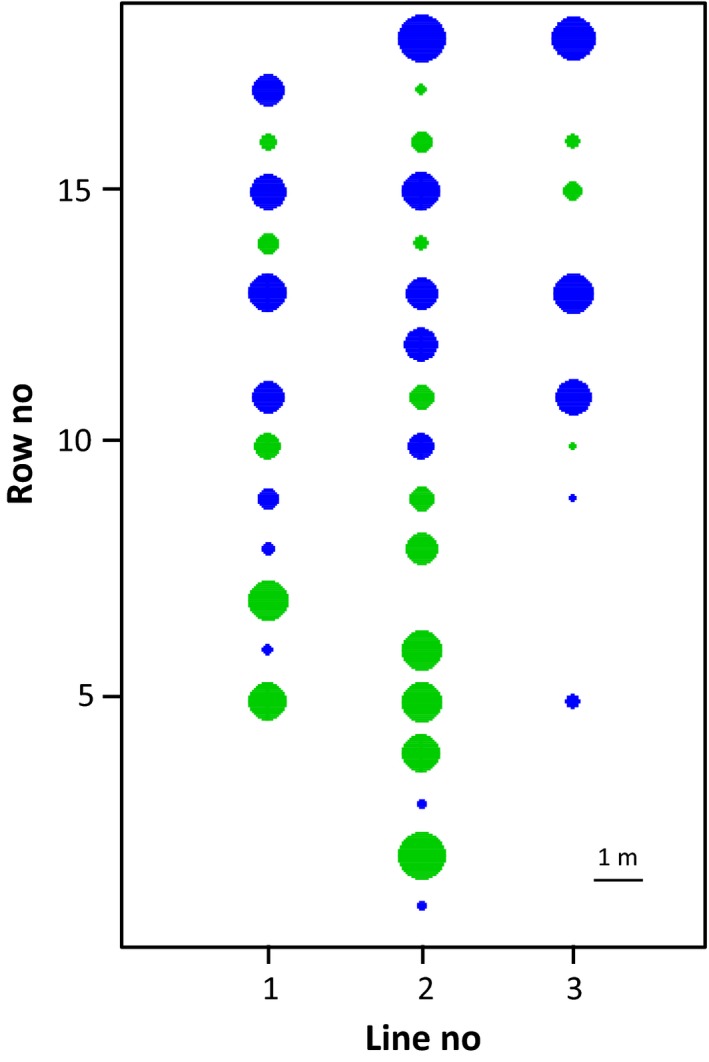
Position of individuals in the experimental plot (see Table S1 and Figure S4) according to their mating group: group A in blue and group B in green. These two groups were defined on the first axis of the correspondence analysis (Table S3). Diameters of blue circles are proportional to coordinates along the second correspondence analysis axis, while for green circles their size is related to third axis coordinate (see Figure S3). For a given group, circle size similarity between individuals thus represents some similarity in mating pattern. For both groups, distribution of size similarity is not randomly distributed in the plot suggesting strong pollination limitation by distance (see also Figure 3)

Among the 444 embryos assigned to a single father each, we investigated cross‐compatibilities between Laperrine's olive individuals. A total of 174 distinct crosses were observed among the 1,369 possible parental combinations, including selfings (12.7%; Tables 2 and S4). Reciprocal crosses were detected for 46 pairs of parents (i.e., 92 of the detected cross combinations; Table 2). Based on these cross‐compatibilities, we looked for preferential mating between groups of individuals of our population by using a FCA (Figure S3; Table S3). The first axis of this analysis explains 22% of the total inertia and this value drops to 9% for the second and third ones, respectively, suggesting that the main pattern in mating relatedness is well represented by the first axis. Actually, along the first axis, a strong and clear pattern is observed with individuals belonging to two nonoverlapping groups: one (group A; Blue) with individuals sharing the same negative coordinate and a second (group B; Green) with individuals sharing the same positive coordinate (Table S3). The corresponding mating pattern is also strong and simple since members of group A (19 individuals) only mate with those of group B (18 individuals) and vice versa (Table 2); these two groups thus correspond to incompatibility groups. Their distribution in the collection is given in Figure S4. The second axis of the FCA is related to the spatial position of group A individuals in the rectangular experimental plot and describes mostly a gradient along the longest side of the collection (North‐South transect; Figure 2 and S3). Similarly, the third axis of the FCA is related to the spatial position of group B individuals describing again a gradient along the longest side of the collection (Figure 2 and S3). This means that nearby individuals of the same incompatibility group tend to mate with the same individuals of the other group. This result also indicates that pollination efficiency may be limited in the collection.

#### 3.3.2 Paternity analyses and cross‐compatibility between Mediterranean olives of the collection

Among the 46 Mediterranean olive embryos, a single known Mediterranean olive father of the collection was identified for 34 of them (Table S5), while the pollen donor of the 12 remaining embryos was not present in our collection. Again, our observations, although limited, are congruent with the existence of two groups of incompatibility. As “L4‐R15” and “Sabina [L4‐R12]” were respectively attributed to cross‐incompatibility groups G1 and G2 (P. Saumitou‐Laprade and Ph. Vernet, personal communication), it was possible to determine to which groups the 13 other flowering trees belong to: accordingly, four belong to G1, while 11 to G2 (Table S5). “Koroneiki [L4‐R14]” (= “Oit52”) and “Arbequina [L4‐R13]” (= “Oit26”) were assigned to G1, and “Manzanilla de Sevilla [L4‐R11]” (= “Oit1”) to G2, as reported in Saumitou‐Laprade, Vernet, Vekemans, Billiard, et al. (2017).

#### 3.3.3 Paternity analyses of putatively hybrid embryos

Among the 29 embryos resulting from controlled crosses, paternity analyses revealed that 16, indeed, resulted from hybridization between the two studied olive subspecies. The remaining 13 embryos resulted from self‐fertilization in three cultivated olive trees: nine for “Koroneiki [L4‐R14],” three for “L4‐R19,” and one for “L4‐R17” (Table S6). Note that no self‐fertilization was observed in the Laperrine's olive. Based on the 16 hybrids, it was finally possible to attribute a cross‐incompatibility group name (following Saumitou‐Laprade, Vernet, Vekemans, Billiard, et al., 2017) to the 37 mature Laperrine's olive trees (following the color scheme presented before: Green = G1, and Blue = G2).

### 3.4 Limitation by distance of pollination efficiency in the Laperrine's olive collection

**Figure 3:**
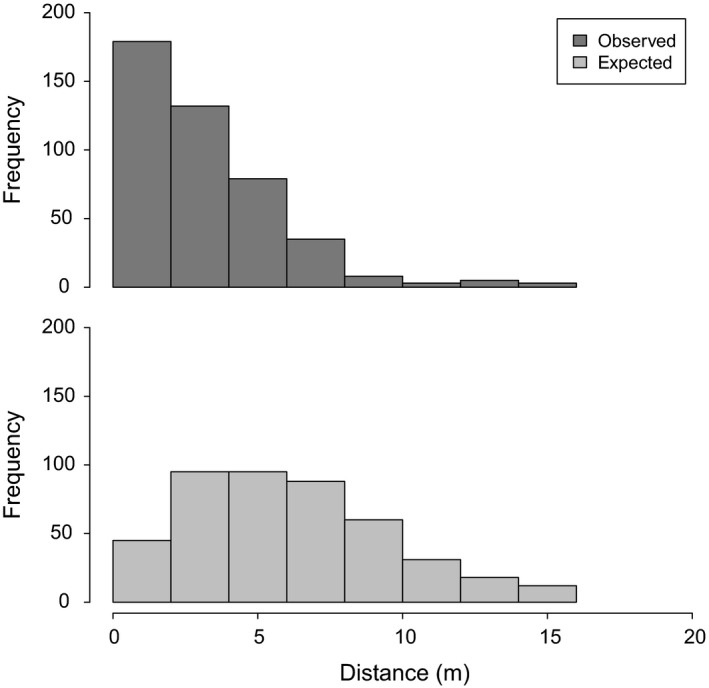
Limitation by distance of the efficient pollination in the Laperrine's olive. Comparison between observed distances of pollination and expected distances considering random crosses between compatible individuals. The expected mean distribution of distances was estimated from the random sampling of 444 embryos, with 1,000 independent iterations

The identification of cross‐incompatibility groups then allowed us to investigate the limitation by distance of pollination efficiency. The observed distance of pollination between Laperrine's olive trees is on average 3.29 m ± 2.51 (Table S7). This result contrasts with the expected mean distance of pollination at random which was estimated at 5.85 m ± 3.42. A highly significant difference between the expected randomly sampled distribution of pollination distance and the observed distribution was revealed (Mann–Whitney *U* test, *V* = 14,195, *p* < 2.2e^−16^). The same pattern is observed when individuals belonging to each group are treated separately, and difference between expected and observed pollination distances remains highly significant (*p* < 2.2e^−16^) in both cases (Figure S5). An excess of short‐distances pollination is observed as the majority of crosses (70.1%) are realized between trees distant less than five meters (Figure 3). As a consequence, embryos are frequently sired by the nearest compatible individual (40.3% of the total number of crosses; Table S7).

## 4 DISCUSSION

Self‐incompatibility system is traditionally assessed in olive using stigma tests or by recording fruit sets in controlled crosses (Farinelli et al., 2018; Saumitou‐Laprade, Vernet, Vekemans, Castric, et al., 2017). Here, we determined cross‐compatibilities with paternity tests among synchronously flowering wild olives in open‐pollinated conditions, confirming the great potential of this alternative approach for investigating the self‐incompatibility genetic determinism (e.g., Arbeiter, Jakse, & Bandelj, 2014; Díaz, Martín, Rallo, Barranco, & de la Rosa, 2006; Montemurro, Dambruoso, Bottalico, & Sabetta, 2019; Mookerjee et al., 2005; Seifi, Guerin, Kaiser, & Sedgley, 2012). The high proportion of seeds (97.8%) assigned to a single father of the Laperrine's olive collection shows that the markers used are sufficiently polymorphic, even with a large number of closely related individuals (full and half siblings). Finally, by determining the father of each seed, the paternity test approach also allows the analysis of other mating features, such as distance of pollination or contribution of each individual to the mating event.

### 4.1 On the diallelic self‐incompatibility system (DSI) in olives

Our analysis, first, empirically supports the existence of two groups of incompatibility in the Laperrine's olive population, as expected under the DSI hypothesis of Saumitou‐Laprade, Vernet, Vekemans, Billiard, et al. (2017). Individuals assigned to an incompatibility group thus always cross with individuals assigned to the other group (Table 2; Figure 2). As a consequence, we also observed numerous reciprocal crosses (46; Table 2). Phenotyping cross‐compatibilities in the Laperrine's olive collection is a prerequisite step before the genetic characterization of the DSI in this taxon, since comparing genomes of the two identified groups of incompatibility will allow locating and help at identifying the genetic factors involved in this important agronomical trait. Similarly to the Laperrine's olive, the few observed cross‐compatibilities among Mediterranean olives did not challenge the DSI hypothesis, but our study did not focus on this subspecies and our observations should be still considered as preliminary.

Second, our results showed no breakdown in the within‐individual SI system in the Laperrine's olive, while variations were observed in the SI system of the Mediterranean olive varieties. In the Laperrine's olive, only one aberrant nonfertilized embryo (either haploid or di‐haploid) was revealed among the 455 seeds analyzed. In addition, no self‐fertilized seed was observed in bags used for controlled crosses for ten mother trees, suggesting these trees are mostly self‐incompatible [but see Besnard et al. (2009) who reported three putative self‐crosses among 212 seeds from ten mother trees of Laperrine's olive]. In contrast, a few cases of self‐fertilization were observed in bags used for controlled crosses on three Mediterranean olive varieties (“Koroneiki [L4‐R14],” “L4‐R17,” and “L4‐R19”). These observations confirm that some cultivated varieties can self‐cross in some conditions (e.g., Androulakis & Loupassaki, 1990; Bartolini & Guerriero, 1995; Fernàndez‐Bolanòs & Frìas, 1969; Ilarioni & Proietti, 2014; Koubouris, Breton, Metzidakis, & Vasilakakis, 2014; Marchese et al., 2016; Moutier, 2000; Wu, Collins, & Sedgley, 2002). This phenomenon has however been referred as leaky self‐incompatibility (Saumitou‐Laprade, Vernet, Vekemans, Billiard, et al., 2017) or pseudo‐self‐compatibility (Alagna et al., 2019), and may occur for some genotypes when flowers meet pollen limitation (particularly when confined in bags) or in peculiar environmental conditions. Here, we observed self‐fertilization on one cultivar of the G2 group and two cultivars of the G1 group [in particular “Koroneiki,” confirming previous reports of selfing on this cultivar (Androulakis & Loupassaki, 1990; Koubouris et al., 2014; Marchese et al., 2016)]. We however did not use stigma tests to investigate the behavior of the self‐pollen on these three cultivars, preventing us to conclude on the reason of self‐crosses (i.e., self‐compatibility mutation vs. leaky self‐incompatibility). Selfing in cultivated olives could indeed result from the artificial selection of self‐compatible mutants over millennia, particularly via their vegetative propagation (Manrique et al., 2019; McKey, Elias, Pujol, & Duputié, 2010; Rowlands, 1964). Recurrent admixture events between divergent olive gene pools (East vs. West; Besnard, Terral, & Cornille, 2018) may also result in a huge phenological variation in the mating system of the cultivated olive, and more frequent selfing is expected in admixed individuals (as shown for instance in hybrids of ash trees and beets; Arnaud, Fénart, Cordellier, & Cuguen, 2010; Gérard, Klein, Austerlitz, Fernández‐Manjarrés, & Frascaria‐Lacoste, 2006). This context could also explain contrasted results reported on the genetic determinism of self‐incompatibility in Mediterranean olives (Farinelli et al., 2018; Saumitou‐Laprade, Vernet, Vekemans, Castric, et al., 2017).

Self‐incompatibility has been considered for a long time as a mechanism avoiding crosses between related individuals, thus preventing inbreeding depression (Darwin, 1876; East, 1940; de Nettancourt, 1977). However, only a few studies have investigated inbreeding depression in self‐incompatible species mainly because of the difficulty to obtain inbred genotypes in such species (Cheptou, Imbert, Lepart, & Escarré, 2000; Porcher & Lande, 2005). Some authors also argued that SI increases the mutation load because recessive lethal mutations are less purged than in self‐compatible species (Lande & Schemske, 1985). The DSI system described in different members of the Oleeae tribe is consistent with an *S*‐locus bearing the dominant allele *S2* and the recessive allele *S1*, leading to the two incompatibility groups G1 and G2 (*S2S1* and *S1S1*, respectively; Billiard et al., 2015). Under such a SI system, the mate availability will be minimal (50%), whereas in multiallelic gametophytic or sporophytic SI systems the proportion of compatible matings in a population will increase with the number of alleles (Vekemans, Schierup, & Christiansen, 1998). The DSI also allows as many or fewer compatible mates among progenies (50%) than other SI systems, except the sporophytic SI with dominance. Yet, a high frequency of crosses between full or half siblings was observed in our study (Figure 1), but this should be mainly due to the nonrandom disposition of related trees in the collection (Figure 1, Table S1) associated to a limitation of pollination by distance (see below). While a DSI system should generally limit the purge of recessive lethal mutations in natural populations, the possibility of reproduction between relatives in small populations should still allow reducing the mutation load, as for instance in invasive populations that were funded on a very limited number of individuals (e.g., <10 in Hawaii; Besnard et al., 2014).

An homomorphic DSI system is shared by distantly related Oleeae species (i.e., *Olea*, *Phillyrea,* and *Fraxinus*; Saumitou‐Laprade, Vernet, Vekemans, Billiard, et al., 2017), but its origin still needs to be better documented in the Oleaceae family. Indeed, the mating system of its ancestor remains unknown as no study has investigated cross‐compatibilities in the four nonpolyploid Oleaceae tribes (i.e., Jasmineae, Forsythieae, Fontanesieae, and Myxopyreae; Wallander & Albert, 2000), for which the homomorphic DSI is thus not documented. Heterostyly (or distyly), that also involves a diallelic system (=heteromorphic DSI), has however been reported in Jasmineae (Olesen, Dupont, Ehlers, Valido, & Hansen, 2005; Thompson & Dommée, 2000), Myxopyreae (Kiew, 1984) and Forsythieae (Hong & Han, 2002; Kim, 1999; Ryu, Yeam, Kim, & Kim, 1976), as well as in *Schrebera* that belongs to the lineage sister to all other Oleeae (Green, 2004; Olofsson et al., 2019). The link between the putative loss of heterostyly with DSI evolution as well as their genetic determinism thus needs to be investigated. The possible role of the whole genome duplication in the Oleeae ancestor also needs to be clarified.

### 4.2 Applications in agronomy and for the management of olive genetic resources

The DSI system in olive implies that half of the trees cannot interbreed (Saumitou‐Laprade, Vernet, Vekemans, Billiard, et al., 2017), which could be a serious limitation for fruit production, especially in modern orchards where a few genotypes are cultivated. Our study shows that microsatellites are efficient in phenotyping cross‐compatibilities and so can be used as a simple test for identifying pollen donors of varieties (Montemurro et al., 2019; Mookerjee et al., 2005). The knowledge on the incompatibility groups can help guiding the assemblage of individuals in the orchard for maximizing pollination, but compatible phenology of varieties for blooming, the possibility of self‐pollination of some cultivars, as well as other features of the site (i.e., topography and prevailing winds), also need to be carefully considered.

Long‐distance pollen dispersals have been reported in natural populations of olive trees (>3 km), but relatively high differences in mean pollination distance were observed between sites, depending, especially, on the topography or positioning of mature, compatible individuals (Beghè, Piotti, Satovic, de la Rosa, & Belaj, 2017; Besnard et al., 2009; Kassa, Konrad, & Geburek, 2018). In the specific conditions of our 1‐year experiment (i.e., blooming during early summer, with limited wind in a high‐density orchard), we observed a highly significant reduction of the pollination distance within the Laperrine's olive collection compared to a random process (3.29 vs. 5.85 m on average; Figure 3), with ca. 40% of crosses done with the nearest compatible individual. Such a pollination limitation by distance could result from the dilution of the pollen cloud from the source father tree. Such mechanisms that affect gene flow are of great importance for the in situ conservation of endangered populations and for the management of ex situ collections. In the wild, crosses between compatible individuals can be indeed limited in fragmented and low‐density populations that may result in preferential mating between some genotypes (Beghè et al., 2017; Besnard et al., 2009; Kassa et al., 2018). In a nursery orchard that aims to produce seeds, the assemblage of individuals should be also carefully thought in order to avoid the production of high levels of inbreeded seedlings (as shown in the present study).

Wild olives are recognized as an important source of genetic variability, which may be valuable in order to enrich the gene pool of cultivated olives and avoid the risk of genetic erosion (Cáceres, Ceccarelli, Pupilli, Sarri, & Mencuccini, 2015; Lavee, Taryan, Levin, & Haskal, 2002; León, de la Rosa, Velasco, & Belaj, 2018). Ongoing climate change raises the need of breeding programs to exploit this wild gene pool, especially to improve drought tolerance but also to prevent the emergence of new pests and diseases. Given that olive oil quality depends on genetic and environmental features, wild olives may be also a resource to improve oil quality traits as oil health value and taste (Baccouri et al., 2011; León et al., 2018). The Laperrine's olive is one of the four wild diploid subspecies known to be a primary genetic resource for the Mediterranean olive (Besnard et al., 2012; Green, 2002), and the production of hybrids shows that the introgression of specific traits from this taxon to the cultivated gene pool is possible (see also Besnard et al., 2013). The knowledge on the incompatibility groups in a collection will greatly facilitate the choice of individuals for controlled crosses, by indicating which pairs of trees cannot be crossed. This will be a potential great gain of time by avoiding a high amount of work due to incompatibility, especially when controlled crosses need to be done with pollen collection conserved at −80°C on successive years.

## CONFLICT OF INTEREST

The authors declare that the research was conducted in the absence of any commercial or financial relationships that could be construed as a potential conflict of interest.

## AUTHOR CONTRIBUTIONS

All authors contributed significantly to the work: G.B. designed the study; G.B, P.O.C., and D.B.C. participated to collecting plants and managing the collection at the CEFE Montpellier; G.B. and M.D. performed the genotyping and data analyses, with the help of P.L., B.H. and J.D. G.B., M.D., and J.D. wrote the manuscript with the help of all co‐authors.

## Data Availability

All relevant data are within this paper and its Supporting Information files.
