# Supplementary material for: Paternity tests support a diallelic self‐incompatibility system in a wild olive (Olea europaea subsp. laperrinei, Oleaceae)
Source: Ecol Evol. 2020 Feb 5;10(4):1876–88. doi: 10.1002/ece3.5993 (PMC7042767; doi:10.1002/ece3.5993)
Supplement: Supplementary file 1 [file ECE3-10-1876-s001.docx]

**Supporting Information**

**Paternity tests support a di-allelic self-incompatibility system in a wild olive (*Olea europaea* subsp. *laperrinei*, Oleaceae)**

Besnard G., *et al.*

**Supporting information includes:**

**TABLE S1.** Plan of the CEFE collection

**TABLE S2.** Summary of knowledge on the 51 Laperrine's olive individuals of the CEFE collection

**TABLE S3.** Summary of the factorial correspondance analysis

**TABLE S4.** Parentage summary of the 455 Laperrine's olive embryos

**TABLE S5.** Parentage summary of the 46 Mediterranean olive seeds genotyped with microsatellites

**TABLE S6.** Parentage summary of the 29 seeds obtained from controlled crosses between Mediterranean and Laperrine’s olives

**TABLE S7.** Summary of the contribution of each Laperrine's olive individual as a father with distance of pollination

**FIGURE S1.** Range distribution of the Laperrine's olive and localities in the Hoggar from where individuals of the CEFE collection were collected

**FIGURE S2.** Mean number of distinct compatible fathers identified per mother tree according to the number of embryos genotyped

**FIGURE S3.** Relationship between individuals coordinates along the second and third axes of the correspondence analysis and position in the experimental plot

**FIGURE S4.** Distribution of cross-incompatibility groups in the Laperrine's olive collection

**FIGURE S5.** Comparison of observed and expected distances of pollination for individuals belonging to group G1 or group G2

**DATA S1.** Microsatellite genetic profiles of individuals characterized in the present study

**REFERENCES**

**TABLE S1.** Plan of the CEFE collection. Individuals in bold flowered in 2018. Individuals underlined are juvenile, while other trees (non bold) are not flowering in 2018 (immature or castrated).

|  | **Row/Line** | **L01** | **L02** | **L03** | **L04** |
| --- | --- | --- | --- | --- | --- |
|  | **R25** |  |  |  | **L4-R25* - 'Al Ascharinah 9' (oleaster)** |
|  | **R24** |  |  |  | **L4-R24* - 'Urla 6'** |
|  | **R23** |  |  |  | **L4-R23 - 'La Repentence 5' (oleaster)** |
|  | **R22** |  |  |  | **L4-R22 - 'Roquebrun 1' (oleaster, feral)** |
|  | **R21** |  |  |  | **L4-R21 - 'Colombale'** |
|  | **R20** |  |  |  | **L4-R20* - 'Amygdalolia'** |
|  | **R19** |  |  | Tizouadj_2_S1 | **L4-R19*** |
|  | **R18** | Adjellela_6_S2 | **Tin-Hamor_1_S1** | **Tonget_A** | L4-R18 - 'BMTO1' (Feral) |
|  | **R17** | **Adjellela_9_S1*** | **Tin-Hamor_1_S2** | Tonget_B | **L4-R17*** |
|  | **R16** | **Adjellela_9_S4** | **Tin-Hamor_1_S4*** | **Tonget_C*** | **L4-R16 - 'Rouget des Alpes de Haute Provence"** |
|  | **R15** | **Adjellela_10_S1*** | **Tin-Hamor_1_S3** | **Tonget_D** | **L4-R15* (G1)** |
|  | **R14** | **Adjellela_10_S2** | **Tin-Hamor_1_S5** | Tonget_E | **L4-R14* - 'Koroneiki'** |
|  | **R13** | **Adjellela_10_S3** | **Tin-Hamor_1_S6*** | **Tonget_F*** | **L4-R13* - 'Arbequina'** |
|  | **R12** | Adjellela_10_S5 | **Tin-Hamor_1_S7** | Tonget_G | **L4-R12* - 'Sabina' (G2)** |
|  | **R11** | **Adjellela_10_S6** | **Tin-Hamor_1_S8** | **Tonget_H** | **L4-R11 - 'Manzanilla de Sevilla'** |
|  | **R10** | **Adjellela_10_S7*** | **Tin-Hamor_1_S9** | **Hadriane_2.1 (3n)** | **L4-R10* - 'Zard'** |
|  | **R9** | **Adjellela_10_S8*** | **Tin-Hamor_1_S10** | **Tonget_3_S1** | *O. e. cuspidata* - Camp27 |
|  | **R8** | **Adjellela_10_S9*** | **Tin-Hamor_1_S11** | Tonget_3_S4 | *O. e. cuspidata* - Camp28 |
|  | **R7** | **Adjellela_10_S10** | Tin-Hamor_1_S12 | Tonget_2_S8 | *O. e. cuspidata* - Camp29 |
|  | **R6** | **Adjellela_10_S11** | **Tin-Hamor_1_S13** | Tonget_2_S10 | *O. e. cuspidata* - Gra3 |
|  | **R5** | **Adjellela_10_S12** | **Tin-Hamor_1_S14*** | **Tonget_2_S11** | *O. e. cuspidata* - Gra7 |
|  | **R4** | *O. e. maroccana -* m7 | **Tin-Hamor_1_S15** | Tonget_1_S1 | *O. e. cuspidata* - Gra5 |
|  | **R3** | *O. e. maroccana -* m8 | **Tin-Hamor_12_S1** | Tonget_1_S4 | *O. e. cuspidata* - Gra8 |
|  | **R2** | *O. e. maroccana -* m9 | **Akerakar_3_S1** | Tonget_1_S5 | *O. e. laperrinei* L1 x *cuspidata* PV1 (Hyb1) |
|  | **R1** | *O. e. cerasiformis -* Cer3 | **Akerakar_3_S2** | Tonget_1_S8 | *O. e. laperrinei* L1 x *cuspidata* PV1 (Hyb2) |

* trees used for hybrid experiments

**TABLE S2.** Summary of knowledge on the 51 Laperrine's olive individuals of the CEFE collection: parents identified in the field based on paternity analyses (Besnard *et al.*, 2009), state of the tree in 2018 (J = juvenile; MT = Mature Tree; IT = Immature Tree), presence of flowers (Fl) or fruits (Fr) from 2013 to 2018, with fruit set in 2018 (+: < 20 fruits; ++: 20 to 50 fruits; +++: > 100 fruits), and observed contribution of each individual as a father (for the 444 seeds assigned to a single tree of the collection; see Table 2). Note that all trees with a limited fruit set (< 50 fruits) also had a limited contribution in the pollination of other trees (siring from 0 to 4 embryos).

**TABLE S3.** Summary of the factorial correspondance analysis (FCA). Coordinates on the three first axes of the FCA are given for each Laperrine’s olive individual. The first axis (explaining 22% of the variance) strongly supports the distinction of two groups (Grp), namely A and B, that were then identified as incompatibility groups (individuals never crossing together; see Table 2). The second and third axes (explaing 9% each) allow revealing variability among groups A and B, respectively. See Figures 3 and S3 for the relationship between coordinates on axes 2 and 3 and position of each tree in the collection.

| **Individual** |  | **Axis 1** | **Grp** |  | **Axis 2** | **Axis 3** |
| --- | --- | --- | --- | --- | --- | --- |
| Adjelella_9_S4 |  | 1 | B |  | 0.032 | -0.752 |
| Adjelella_10_S2 |  | 1 | B |  | 0.017 | -0.400 |
| Adjelella_10_S7 |  | 1 | B |  | -0.004 | 0.085 |
| Adjelella_10_S10 |  | 1 | B |  | -0.057 | 1.335 |
| Adjelella_10_S12 |  | 1 | B |  | -0.050 | 1.163 |
| Tin-Hamor_1_S2 |  | 1 | B |  | 0.054 | -1.266 |
| Tin-Hamor_1_S4 |  | 1 | B |  | 0.020 | -0.469 |
| Tin-Hamor_1_S5 |  | 1 | B |  | 0.039 | -0.916 |
| Tin-Hamor_1_S10 |  | 1 | B |  | 0.003 | -0.080 |
| Tin-Hamor_1_S11 |  | 1 | B |  | -0.027 | 0.645 |
| Tin-Hamor_1_S13 |  | 1 | B |  | -0.056 | 1.315 |
| Tin-Hamor_1_S14 |  | 1 | B |  | -0.052 | 1.232 |
| Tin-Hamor_1_S15 |  | 1 | B |  | -0.047 | 1.113 |
| Akerakar_3_S1 |  | 1 | B |  | -0.087 | 2.047 |
| Tonget_C |  | 1 | B |  | 0.044 | -1.035 |
| Tonget_D |  | 1 | B |  | 0.025 | -0.597 |
| Tin-Hamor_1_S8 |  | 1 | B |  | 0.005 | -0.112 |
| Hadriane_2.1 |  | 1 | B |  | 0.074 | -1.738 |
| Adjelella_9_S1 |  | -1 | A |  | 0.422 | 0.018 |
| Adjelella_10_S1 |  | -1 | A |  | 0.718 | 0.031 |
| Adjelella_10_S3 |  | -1 | A |  | 0.866 | 0.037 |
| Adjelella_10_S6 |  | -1 | A |  | 0.483 | 0.021 |
| Adjelella_10_S8 |  | -1 | A |  | -0.418 | -0.018 |
| Tin-Hamor_1_S1 |  | -1 | A |  | 1.692 | 0.072 |
| Tin-Hamor_1_S3 |  | -1 | A |  | 0.886 | 0.038 |
| Tin-Hamor_1_S6 |  | -1 | A |  | 0.490 | 0.021 |
| Tonget_A |  | -1 | A |  | 1.411 | 0.060 |
| Tonget_F |  | -1 | A |  | 1.125 | 0.048 |
| Tonget_H |  | -1 | A |  | 0.736 | 0.031 |
| Tin-Hamor_1_S7 |  | -1 | A |  | 0.612 | 0.026 |
| Tin-Hamor_1_S9 |  | -1 | A |  | 0.073 | 0.003 |
| Adjelella_10_S9 |  | -1 | A |  | -0.931 | -0.040 |
| Adjelella_10_S11 |  | -1 | A |  | -1.175 | -0.050 |
| Tin-Hamor_12_S1 |  | -1 | A |  | -1.275 | -0.054 |
| Akerakar_3_S2 |  | -1 | A |  | -1.325 | -0.056 |
| Tonget_3_S1 |  | -1 | A |  | -1.501 | -0.064 |
| Tonget_2_S11 |  | -1 | A |  | -0.803 | -0.034 |

**TABLE S4**. Parentage summary of the 455 Laperrine's olive embryos. Mothers are in rows, fathers in columns. Pairs of reciprocal crosses are framed in bold line. A total of 445 embryos were assigned to a single father (444 if we exclude the non fertilized embryo). Blue and green colors were arbitrarily used to distinguish individuals from the two identified groups (*a posteriori* corresponding to G2 and G1, respectively; see Table S3).

Overall, we recorded 250 "G2 x G1" embryos (from 90 distinct crosses involving 19 G2 mothers and 16 G1 fathers) and 194 "G1 x G2" embryos (from 84 distinct crosses involving 17 G1 mothers and 17 G2 fathers)

^a^ assigned to at least two putative fathers of the collection (8 embryos), or a tree from outside the collection (1 embryo); ^b^ non-fertilized embryo (haploid or di-haploid).

**TABLE S5.** Parentage summary of the 46 Mediterranean olive seeds genotyped with microsatellites.

Mothers are in rows, fathers in columns. Note that L4-R12 ('Sabina') and L4-R15 were respectively assigned to groups G2 and G1 (Saumitou‐Laprade *et al.*, 2017). Blue and green colors are here used to distinguish individuals between these putative incompatibility groups (blue for G2 individuals and green for G1 individuals).

|  |  | **Father** | | | | | | | | | | | | | | | |  |
| --- | --- | --- | --- | --- | --- | --- | --- | --- | --- | --- | --- | --- | --- | --- | --- | --- | --- | --- |
|  |  | [L4-R10] | [L4-R11] | **[L4-R12]^G2^** | [L4-R13] | [L4-R14] | **[L4-R15]^G1^** | [L4-R16] | [L4-R17] | [L4-R19] | [L4-R20] | [L4-R21] | [L4-R22] | [L4-R23] | [L4-R24] | [L4-R25] | Others* | **Total** |
| **Mother** | **[L4-R10]** |  |  |  |  | 2 | 1 |  |  |  |  |  |  |  |  |  |  | 3 |
|  | [L4-R11] |  |  |  | 1 | 1 |  |  |  |  |  |  |  |  |  |  | 1 | 3 |
|  | **[L4-R12]^G2^** |  |  |  | 3 |  |  |  |  |  |  |  |  |  |  |  |  | 3 |
|  | [L4-R13] |  |  |  |  |  |  |  | 3 |  |  |  |  |  |  |  |  | 3 |
|  | [L4-R14] |  |  | 1 |  |  |  |  | 2 |  |  |  |  |  |  |  |  | 3 |
|  | **[L4-R15]^G1^** |  |  | 1 |  |  |  |  | 3 |  |  |  |  |  |  |  |  | 4 |
|  | [L4-R16] |  |  |  |  | 1 | 1 |  |  |  |  |  |  |  |  |  | 1 | 3 |
|  | [L4-R17] |  |  |  |  |  |  |  |  |  |  |  |  |  |  |  | 3 | 3 |
|  | [L4-R19] |  |  |  |  |  |  |  | 2 |  |  | 1 |  |  |  |  |  | 3 |
|  | [L4-R20] |  |  |  |  |  |  |  |  | 3 |  |  |  |  |  |  |  | 3 |
|  | [L4-R21] |  |  |  |  |  | 1 |  |  |  |  |  |  |  |  |  | 2 | 3 |
|  | [L4-R22] |  |  |  | 1 |  | 1 |  |  |  |  |  |  |  |  |  | 1 | 3 |
|  | [L4-R23] |  |  |  | 1 |  | 1 |  |  |  |  |  |  |  |  |  | 1 | 3 |
|  | [L4-R24] |  |  |  | 1 |  |  |  |  |  |  |  |  |  |  |  | 2 | 3 |
|  | [L4-R25] |  |  |  |  | 1 | 1 |  |  |  |  |  |  |  |  |  | 1 | 3 |
|  | **Total** | 0 | 0 | 2 | 7 | 5 | 6 | 0 | 10 | 3 | 0 | 1 | 0 | 0 | 0 | 0 | 12 | **46** |

* : embryos with an unknown father (from outside the collection).

**TABLE S6.** Parentage summary of the 29 seeds obtained from controlled crosses between Mediterranean and Laperrine’s olives.

Mothers are in rows, fathers in columns. Blue and green colors are arbitrarily used to distinguish individuals from the two incompatibility groups (blue for G2 individuals and green for G1 individuals; see Table S5). Sixteen embryos are from hybridization between Laperrine’s olives and Mediterranean olives, while the remaining 13 embryos are self-progenies of three cultivated olive varieties.

|  |  | **Father** | | | | | | | | |  |
| --- | --- | --- | --- | --- | --- | --- | --- | --- | --- | --- | --- |
|  |  | [L4-R11] | [L4-R14] | [L4-R17] | [L4-R19] | [L4-R20] | Tin-Hamor_1_S4 | Tin-Hamor_1_S14 | Adjelella_10_S7 | Adjellela_10_S9 | **Total** |
| **Mother** | [L4-R11] ('Manzanilla de Sevilla') |  |  |  |  |  |  |  |  |  | 0 |
|  | [L4-R14] ('Koroneiki') |  | **7*** |  |  |  |  |  |  | 6 | 13 |
|  | [L4-R17] |  |  | **1*** |  |  |  |  |  |  | 1 |
|  | [L4-R19] |  |  |  | **5*** |  |  |  |  |  | 5 |
|  | [L4-R20] ('Amygdalolia') |  |  |  |  |  |  |  |  |  | 0 |
|  | Tin-Hamor_1_S4 |  |  | 1 |  |  |  |  |  |  | 1 |
|  | Tin-Hamor_1_S14 | 5 |  | 2 |  |  |  |  |  |  | 7 |
|  | Adjelella_10_S7 |  |  |  |  | 2 |  |  |  |  | 2 |
|  | Adjellela_10_S9 |  |  |  |  |  |  |  |  |  | 0 |
|  | **Total** | 5 | 7 | 4 | 5 | 2 | 0 | 0 | 0 | 6 | 29 |

* : crosses resulting from self-fertilization.

**TABLE S7.** Summary of the contribution of each mature Laperrine's olive individual as a father with distance of pollination. N_emb_ = Number of embryos sired by the father; Nearest = Number of embryos which result from a cross with the nearest compatible mother; Others = Number of embryos which not result from a cross with the nearest compatible mother.

| **Father** |  | **N_emb_** |  | **Compatible mother** | |  | **Distance (m)** | |
| --- | --- | --- | --- | --- | --- | --- | --- | --- |
|  |  |  |  | **Nearest** | **Others** |  | **Mean** | **SD** |
| Adjellela_9_S1 |  | 19 |  | 7 | 12 |  | 4.05 | 3.75 |
| Adjellela_9_S4 |  | 22 |  | 11 | 11 |  | 2.96 | 2.21 |
| Adjellela_10_S1 |  | 10 |  | 6 | 4 |  | 2.16 | 1.72 |
| Adjellela_10_S2 |  | 22 |  | 14 | 8 |  | 1.83 | 1.2 |
| Adjellela_10_S3 |  | 7 |  | 3 | 4 |  | 3.18 | 2.05 |
| Adjellela_10_S6 |  | 16 |  | 9 | 7 |  | 2.83 | 2.4 |
| Adjellela_10_S7 |  | 66 |  | 29 | 37 |  | 3.05 | 2.37 |
| Adjellela_10_S8 |  | 27 |  | 3 | 24 |  | 3.87 | 1.9 |
| Adjellela_10_S9 |  | 32 |  | 10 | 22 |  | 2.78 | 1.68 |
| Adjellela_10_S10 |  | 1 |  | 1 | 0 |  | 1.00 | NA |
| Adjellela_10_S11 |  | 10 |  | 4 | 6 |  | 3.14 | 2.95 |
| Adjellela_10_S12 |  | 14 |  | 7 | 7 |  | 2.9 | 2.63 |
| Tin-Hamor_1_S1 |  | 3 |  | 3 | 0 |  | 1.00 | 0 |
| Tin-Hamor_1_S2 |  | 11 |  | 2 | 9 |  | 3.91 | 3.17 |
| Tin-Hamor_1_S3 |  | 16 |  | 10 | 6 |  | 2.3 | 2.56 |
| Tin-Hamor_1_S4 |  | 36 |  | 15 | 21 |  | 3.07 | 2.2 |
| Tin-Hamor_1_S5 |  | 0 |  | - | - |  | - | - |
| Tin-Hamor_1_S6 |  | 14 |  | 4 | 10 |  | 3.1 | 1.44 |
| Tin-Hamor_1_S7 |  | 2 |  | 0 | 2 |  | 3.92 | 0.45 |
| Tin-Hamor_1_S8 |  | 1 |  | 1 | 0 |  | 1.00 | NA |
| Tin-Hamor_1_S9 |  | 1 |  | 1 | 0 |  | 1.00 | NA |
| Tin-Hamor_1_S10 |  | 7 |  | 6 | 1 |  | 1.28 | 0.76 |
| Tin-Hamor_1_S11 |  | 1 |  | 0 | 1 |  | 4.24 | 0 |
| Tin-Hamor_1_S13 |  | 8 |  | 0 | 8 |  | 3.94 | 0.79 |
| Tin-Hamor_1_S14 |  | 23 |  | 4 | 19 |  | 4.03 | 1.52 |
| Tin-Hamor_1_S15 |  | 12 |  | 3 | 9 |  | 3.62 | 3.33 |
| Tin-Hamor_12_S1 |  | 2 |  | 1 | 1 |  | 3.3 | 0.43 |
| Akerakar_3_S1 |  | 1 |  | 1 | 0 |  | 1.00 | NA |
| Akerakar_3_S2 |  | 17 |  | 4 | 13 |  | 4.37 | 4.27 |
| Tonget_2_S11 |  | 4 |  | 0 | 4 |  | 5.07 | 3.82 |
| Tonget_3_S1 |  | 2 |  | 0 | 2 |  | 5.83 | NA |
| Tonget_A |  | 0 |  | - | - |  | - | - |
| Tonget_C |  | 7 |  | 1 | 6 |  | 5.86 | 3.82 |
| Tonget_D |  | 18 |  | 2 | 16 |  | 5.4 | 2.99 |
| Tonget_F |  | 12 |  | 4 | 8 |  | 3.5 | 1.7 |
| Tonget_H |  | 0 |  | - | - |  | - | - |
| Hadriane_2.1 |  | 0 |  | - | - |  | - | - |
| TOTAL |  | 444 |  | 166 | 278 |  | 3.29 | 2.51 |


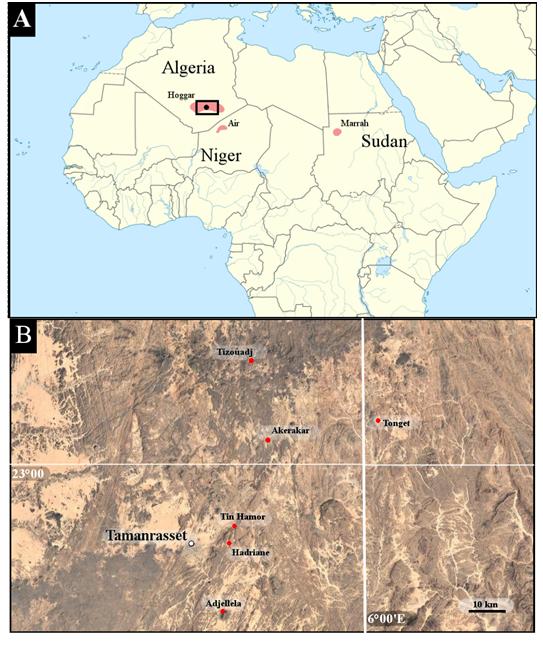


**FIGURE S1.** Range distribution of the Laperrine's olive (A) and localities in the

Hoggar from where individuals of the CEFE collection were collected (B)

**
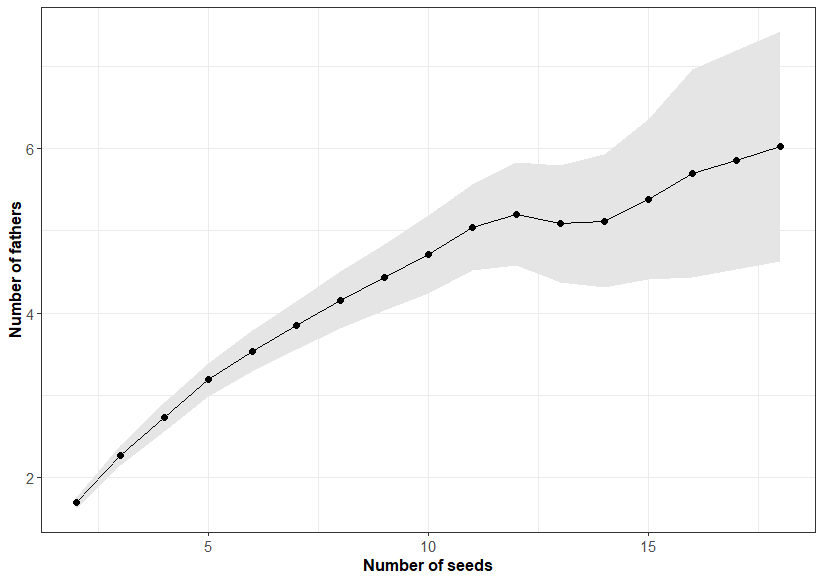
**

**FIGURE S2.** Mean number of distinct compatible fathers identified per mother tree according to the number of embryos genotyped (*K*). For each mother tree, we estimated the mean number of fathers (*n_f_*) identified for *K* embryos using a random sampling without replacement (10'000 iterations).The *n_f_* matrix allowed us to reconstruct an accumulation curve for *K* ranging from 1 to 18. At each value of *K*, we only considered mother trees with at least *K* embryos analyzed (a minimum of six mother trees was considered for *K* = 15 to 18). The grey shade area represents the 95%-confidence interval of the *n_f_* estimate.

**FIGURE S3.** Relationship between individuals coordinates along the second (left) and third (right) axes of the correspondence analysis and position (row) in the experimental plot. Group A and group B are in blue and green, respectively.

**FIGURE S4.** Distribution of cross-incompatibility groups in the Laperrine's olive collection (Green = G1; Blue = G2; White = Unknown; See Table S1 for individual correspondence).

**FIGURE S5.** Comparison of the observed and expected distances of pollination for individuals belonging to group G1 (left) or group G2 (right). The difference remains highly significant (pairwise Wilcoxon-test with Holm correction: P < 2.2e^-16^) in both cases. The same test reveals that the distributions are not significantly different between groups (for both observed and expected distributions; P > 0.5).

**REFERENCES**

Besnard, G., Baali-Cherif, D., Bettinelli-Riccardi, S., Parietti, D., & Bouguedoura, N. (2009). Pollen-mediated gene flow in a highly fragmented landscape: consequences for defining a conservation strategy of the relict Laperrine’s olive. *Comptes Rendus Biologies*, *332*, 662–672.

Saumitou‐Laprade, P., Vernet, P., Vekemans, X., Billiard, S., Gallina, S., Essalouh, L., ... Baldoni, L. (2017). Elucidation of the genetic architecture of self‐incompatibility in olive: Evolutionary consequences and perspectives for orchard management. *Evolutionary Applications*, *10*, 867–880.
